# Supplementary figures and images for: Arsenic Trioxide Triggers Apoptosis of Metastatic Oral Squamous Cells Carcinoma with Concomitant Downregulation of GLI1 in Hedgehog Signaling
Source: Biomedicines. 2022 Dec 19;10(12):3293. doi: 10.3390/biomedicines10123293 (PMC9775978; doi:10.3390/biomedicines10123293)

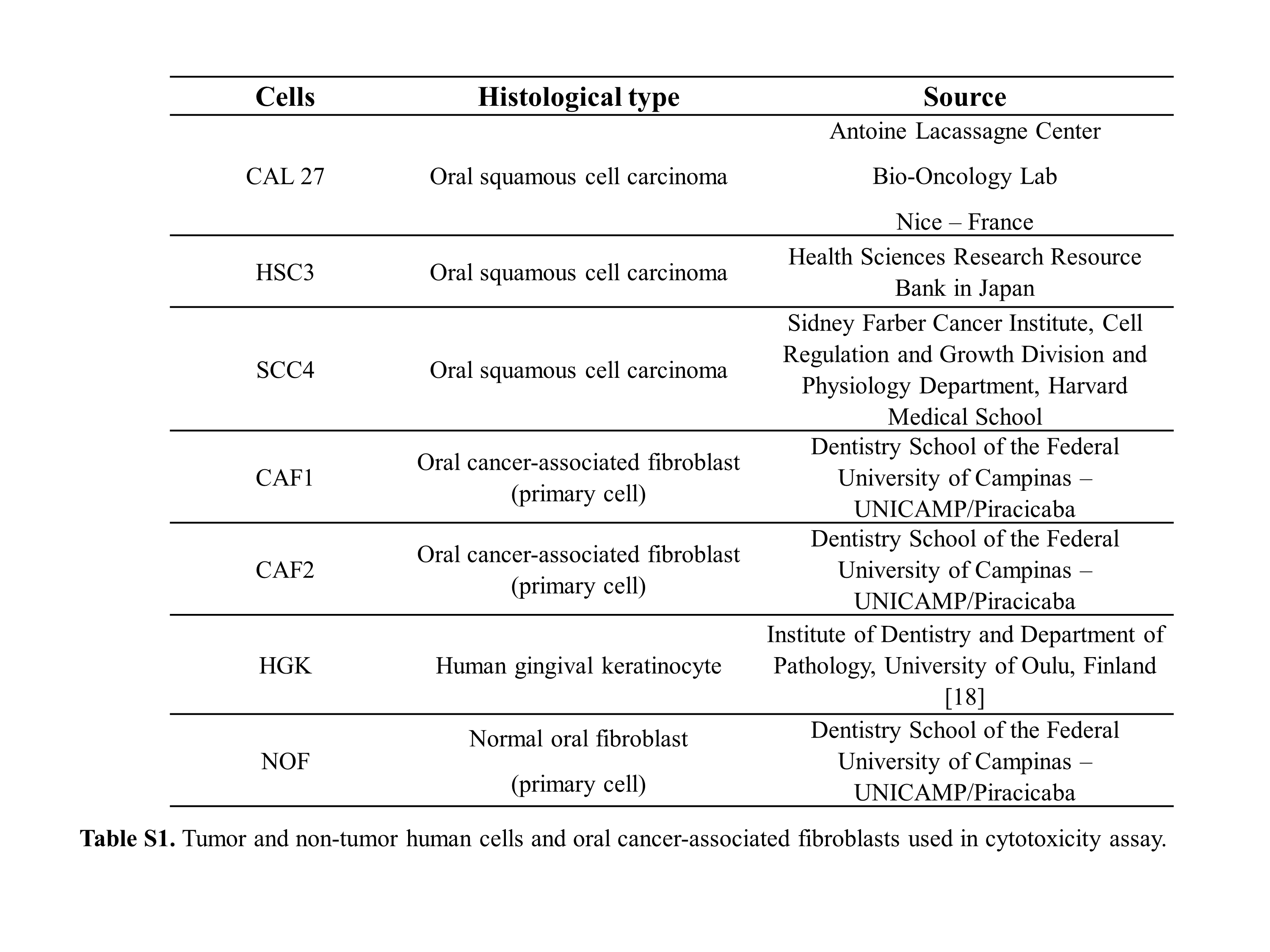

Supplement: Supplementary file 1 [file biomedicines-10-03293-s001.zip › biomedicines-2010942-supplementary.tif]
